# Primary Ovarian Angiosarcoma: Diagnostic Challenges and Conundrums

**DOI:** 10.15190/d.2024.17

**Published:** 2024-12-31

**Authors:** Sujata Agrawal, Zachariah Chowdhury, Roma Jethani

**Affiliations:** ^1^Department of Oncopathology Homi Bhabha Cancer Hospital (HBCH) and Mahamana Pandit Madan Mohan Malviya Cancer Centre (MPMMCC), Tata Memorial Centre, Homi Bhabha National Institute (HBNI), Varanasi, India

**Keywords:** Angiosarcoma, Ovarian, Malignancy, Immunohistochemistry

## Abstract

Angiosarcoma is an extremely uncommon mesenchymal neoplasm overall and moreso in female genital organs such as the ovary. Diagnosing primary ovarian angiosarcoma remains challenging on clinical grounds due to the absence of specific clinical symptoms as well as on histopathological analysis especially in poorly differentiated subtypes due to non-specific and overlapping morphologic features. Misdiagnosis in such scenarios can be devastating as this tumor is clinically very aggressive. We describe a case of primary ovarian angiosarcoma in a 33-year-old multiparous female with bilateral ovarian masses and metastasis at diagnosis. Histopathologic appraisal revealed a poorly differentiated malignant tumor with varied differential diagnoses. The saviour in such a scenario was the immunohistochemistry findings, underlining the incredible utility of this technique in the precise diagnosis and evasion of misdiagnosis. This case accentuates the paramount importance of precise diagnostic modalities in shaping clinical practice and enriching the scientific understanding of rare and aggressive neoplasms. Against this backdrop, the potential pitfalls and pearls while dealing with this entity have been elucidated, along with a review of the recent literature.

## INTRODUCTION

Angiosarcoma, constituting less than 1% of all sarcomas, is an exceptionally rare clinically aggressive vascular tumor, primarily found in the skin and soft tissue ^1^. Nonetheless, it can affect any part of the body and there are documented cases of angiosarcoma affecting visceral organs such as the liver, spleen, heart, gastrointestinal tract, and female genital tract ^2,3^. Within the female genital tract, ovary is a very infrequent site, typically presenting as a solitary occurrence, seldom originating from pre-existing ovarian neoplasms like carcinosarcoma, mature teratomas, desmoid cysts, or epithelial neoplasms. Diagnosing primary ovarian angiosarcoma poses a challenge due to the absence of specific clinical symptoms in patients. Morphologically as well it can be easily confused with common primary ovarian malignant neoplasms leading to misdiagnosis. Herein, we present a case of primary ovarian angiosarcoma in a premenopausal woman, exploring the clinicopathological and immunohistochemical profiles associated with this condition.

## CASE PRESENTATION

**Figure 1:**
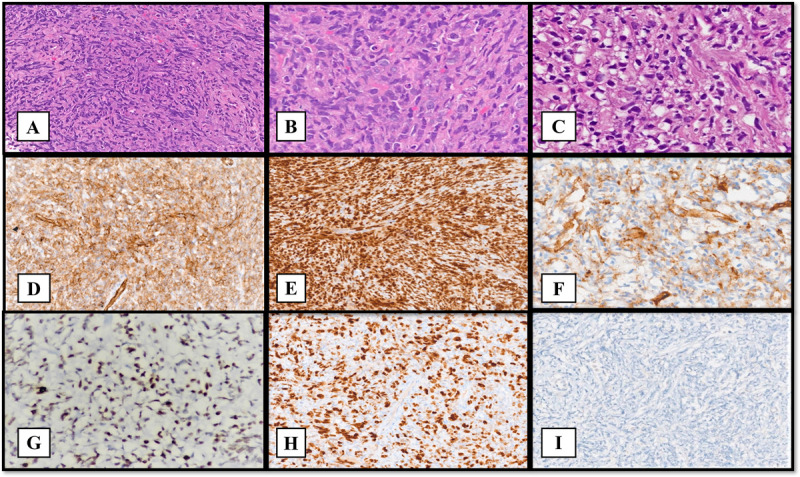
Microscopic images of ovarian angiosarcoma A-B: show an infiltrating tumor disposed in dyscohesive sheets which showed significant pleomorphism and brisk mitosis (10x and 40x respectively). C: Intracytoplasmic vacuoles? Lumina formation (40x). Tumor cells show diffuse immunopositivity for CD31 (D, 10x), ERG (E, 10x), and CD34 (F, 20x). SF-1 shows focal positivity (G, 10x). Ki67 proliferative index is ~80% (H, 10x). Tumor cells show immunonegativity for AE1/AE3 (I,10x).

A 33-year-old multiparous woman presented with mild abdominal pain and distension for 3 months, along with one episode of polymenorrhea. There was no history of weight loss, loss of appetite, vaginal bleeding, or any family history of cancer. Additionally, there was no prior history of exposure to drugs, carcinogens or radiation. On examination, no abnormality was noted, except for the presence of fluid thrill. Tumor markers like serum Alpha-fetoprotein (AFP), Carcinoembryonic antigen (CEA), total Human Chorionic Gonadotropin (HCG), and Carbohydrate antigen (CA) 19.9 were within normal limits. However, Cancer antigen (CA) 125 evinced a slight increase, measuring 98.9 U/ml, and Lactate Dehydrogenase enzyme (LDH) was significantly elevated at 429 U/L. Magnetic Resonance Imaging scan revealed large lobulated heterogeneous signal intensity masses arising from both ovaries, measuring 10 x 15 x 17 cm on the right side, and 9 x 13 x 8 cm on the left side. A similar intensity deposit, measuring approximately 8.2 x 7.7 cm, was observed in the Pouch of Douglas, displaying ill-defined fat planes with the posterior bladder wall and the rectum, involving bilateral medial 2/3rds of the parametrium, and abutting the cervix and uterine body. Additionally, extensive peritoneal disease was also identified. Radiological impression was ovarian carcinoma with metastasis. A biopsy was performed on the right adnexal mass, and the histopathological examination (HPE) revealed fibrocollagenous tissue infiltrated by numerous atypical cells in dyscohesive sheets. The individual cells were oval to spindle shaped and displayed hyperchromatic, pleomorphic, irregular nuclei, inconspicuous nucleoli, and scant cytoplasm with ill-defined borders. Few cells exhibited intracytoplasmic vacuoles. Brisk mitosis (> 20/10 High Power Fields), apoptotic bodies, and scattered red blood cells were noted amidst the tumor cells. Necrosis, native ovarian parenchyma, or any teratomatous elements were not identified in the submitted tissue. On immunohistochemistry (IHC) analysis, the tumor cells revealed strong and diffuse positivity for CD31, CD34, and ERG, and focal positivity for SF1. They were negative for epithelial markers, including PanCK (AE1/AE3), Epithelial Membrane Antigen (EMA), CK7, and CK20, as well as for WT-1, SALL4, calretinin, inhibin, Leucocyte Common Antigen (LCA), CD3 and CD20. Hormone receptor analysis for estrogen receptor and progesterone receptor did not yield positive results. The proliferative index Ki67 was approximately 80%. The diagnosis proffered was ovarian angiosarcoma (Figure 1).

Ascitic fluid evaluation was negative for malignancy. Owing to metastatic disease, the patient was administered chemotherapy comprising Nab. Paclitaxel (300mg) and Bevacizumab (400mg). Post-six cycles of chemotherapy, Computed Tomography scan revealed a reduction in size with a partial response to treatment, and follow-up till date (12 months since diagnosis) documented the patient to be alive with residual disease.

## DISCUSSION

Angiosarcoma (AS) is a rare highly malignant vascular neoplasm usually associated with poor outcomes1. At the clinical level, AS can be primary or secondary to various etiologies (radiation-associated AS, often for breast cancer or less commonly documented with chronic lymphedema). Ovarian angiosarcoma is extremely rare, the incidence of primary ovarian angiosarcoma being 1/1,000,000 with around 40 reported cases in the literature ^2-6^. It mainly occurs in premenopausal women (mean age 33 years old), presenting with abdominal pain and distension, as was the scenario in our case ^7,8^. Unilateral involvement of ovary is more common, a finding dissimilar to the case under study ^1-4^. Histopathological confirmation is indispensable for the precise diagnosis of primary ovarian angiosarcoma. The microscopic appearance of angiosarcoma can vary from focal or diffuse vasoformative components composed of dilated, slit like, papillary, or interconnecting vascular spaces lined by atypical endothelial cells to solid components that may be spindle sarcomatous or epithelioid. Differentiated angiosarcoma typically displays plump, pleomorphic, and mitotically active

**Table 1:** Immunohistochemistry of ovarian angiosarcoma and differential diagnosis. AS: Angiosarcoma, PDC: Poorly differentiated carcinoma, Met. Ca: Metastatic carcinoma, MGCT: Malignant germ cell tumor, HG-ESS: High grade endometrial stromal sarcoma, SCST: Sex cord stromal tumor, NHL: Non-Hodgkin lymphoma, MM: Malignant melanoma, ES: Ewing sarcoma, LPS: Liposarcoma, LMS: Leiomyosarcoma, RMS: Rhabdomyosarcoma, FS: Fibrosarcoma, MPNST: Malignant peripheral nerve sheath tumor, UPS: Undifferentiated pleomorphic sarcoma. a: ERG can be positive in ES with EWSR1-ERG or FUS-ERG rearrangement. b: WT-1 is positive in High-grade serous carcinoma and is variable in Endometrioid carcinoma. c: Desmin can be positive in MPNST with rhabdomyoblastic differentiation. +: all (or nearly all) cases positive. +/ -: variably positive -: all (or nearly all) cases negative

No	IHC markers	AS	PDC/Met.Ca	MGCT	HG-ESS	SCST	NHL	MM	ES	LPS	LMS	RMS	FS	MPNST	UPS
1	CK (AE1/AE3)	+/-	+	+/-	+/-	+/-	-	-	-	-	-	-	-	-	-
2	CD31/ERG	+	-	-	-	-	-	-	-a	-	-	-	-	-	-
3	SALL4	-	-	+	-	-	-	-	-	-	-	-	-	-	-
4	WT-1	-	+b	-	-	+	-	-	-	-	-	-	-	-	-
5	Calretinin/ inhibin	-	-	-	-	+	-	-	-	-	-	-	-	-	-
6	CD10/ CyclinD1	-	-	-	+	-	+	-	-	-	-	-	-	-	-
7	LCA	-	-	-	-	-	+	-	-	-	-	-	-	-	-
8	S100	-	-	-	-	-	-	+	-	+/-	-	-	-	+/-	-
9	NKX2.2	-	-	-	-	-	-	-	+	-	-	-	-	-	-
10	SMA/h-caldesmon	-	-	-	-	-	-	-	-	-	+	-	-	-	-
11	Desmin	-	-	-	-	-	-	-	-	-	+	+	-	-c	-
12	H3K27me3	+	+	+	+	+	+	+	+	+	+	+	+	-	+

tumor cells, that can be spindle-shaped, polygonal, epithelioid, or primitive round cells, forming papillae or solid nests within vascular lumina. In our case, other vascular tumors such as cellular haemangioma and epithelioid/spindle haemangioendothelioma were distinguished based on the non vasoformative solid growth patterns, high cellularity, marked nuclear atypia, brisk mitosis, numerous apoptotic bodies and high Ki67 index. A diagnosis may be onerous if the vascular nature of the neoplasm is not evident and there are more poorly differentiated areas manifesting solid growth patterns, as was observed in our case. The differential diagnoses with such histology include a variety of high grade malignancies such as poorly differentiated carcinoma including high-grade serous carcinoma, high-grade endometrioid carcinoma, and metastatic carcinoma, carcinosarcoma, malignant germ cell tumor, high-grade endometrial stromal sarcoma, sex cord-stromal tumor, lymphoma, melanoma and other high grade sarcomas such as Ewing sarcoma, liposarcoma, leiomyosarcoma, rhabdomyosarcoma, fibrosarcoma, malignant peripheral nerve sheath tumor, and undifferentiated pleomorphic sarcoma. In such context, the incredible role of IHC cannot be understated in the veracious recognition of angiosarcoma (Table 1). CD31 and CD34 are expressed in more than 90% and 50-60% of angiosarcomas respectively. CD31 seems to be a more sensitive and specific immunoantibody than CD34 in case of poorly differentiated angiosarcoma. ERG and FLI1 are relatively sensitive and specific markers of vascular tumors, regardless of the type and level of malignancy ^9,10^. Nevertheless, these can also be expressed in some tumors of nonvascular origin. Thus, combinations of one or more of these vascular markers [CD31, CD34, ERG, and FLI1], along with other immunomarkers are required for prompt and accurate diagnosis of angiosarcoma. Another engaging element in this study which can be underlined is the immunoreactivity of SF1. SF1 can be positive in ovarian tumors other than sex cord

**Table 2:** Summary of outcomes and findings of recent reported cases of primary angiosarcoma of the ovary NA, not available; DOD, dead of disease; AWD, alive with disease; NED, No evidence of disease; TAH, total abdominal hysterectomy; BSO, bilateral salpingo-oophorectomy; RT, radiotherapy; MAID chemotherapy (mesna, adriamycin/doxorubicin, ifosfamide and dacarbazine).

Author (year)[ref]	Age (Years)	Stage	Primary Treatment	Adjuvant Treatment	Follow-up (months)	Status
Yaqoob et al., 2014^5^	41	Ia	Left salpingo-oophorectomy	None	Lost to follow up	NA
Hong Ye et al, 2021^3^	47	Ia	TAH/BSO, staging	RT (15 fractions); Olaparib, immunotherapy	9	NED
Peng et al, 2021^2^	25	Presumed stage I (unstaged, pregnant)	None	None	<1	DOD
Zhou et al, 2023^1^	51	IV	Cytoreduction Surgery	MAID chemotherapy, 1cycle	1	DOD
	41		TAH/BSO	MAID chemotherapy, 6 cycle, anti-PD-1 immunotherapy (nivolumab)	27	NED
Johnson et al, 2023^4^	29	I	Cytoreduction Surgery	Olaparib	20	NED
Current case	33	IV	Chemotherapy	Nab. paclitaxel (300mg) and Bevacizumab (400mg), 6 cycles	12	AWD

stromal tumors and its reactivity should not be viewed in isolation when deliberating on a diagnosis of sex cord stromal tumors. For localized angiosarcoma, surgery remains the mainstay of curative management ^4^. However, due to its aggressive nature for local recrudescence and distant metastases, studies have shown better disease control with postoperative adjuvant chemotherapy or radiotherapy. Treatment for advanced angiosarcoma typically includes systemic chemotherapy, anti-angiogenic agents, and recently, immune checkpoint inhibitors ^3,4,11^. The chemotherapy regimen comprising Nab-paclitaxel and Bevacizumab administered in our patient was somewhat effective, leading to tumor size reduction post six cycles of therapy and survival of the patient on a follow-up period of 12 months (Table 2).

## CONCLUSION

A high index of suspicion and pathologic acumen along with meticulous histopathological and immunohistochemistry analysis, are the cornerstones for accurate diagnosis. Despite aggressive treatment strategies, the high rates of metastasis and recurrence underscore the need for early detection and a multidisciplinary approach. Further research is essential to establish more effective diagnostic and therapeutic protocols for this arduous and recherché clinical condition. These insights hold significant implications in clinical practice by facilitating prompt and accurate intervention while also contributing to the scientific community’s understanding of rare and aggressive neoplasms, ultimately paving the way for improved patient outcomes.
